# Immunohistochemical evaluation of ROCK activation in invasive breast cancer

**DOI:** 10.1186/s12885-015-1948-8

**Published:** 2015-12-01

**Authors:** Chih-Yi Hsu, Zee-Fen Chang, Hsiao-Hui Lee

**Affiliations:** 1 Department of Pathology and Laboratory Medicine, Taipei Veterans General Hospital, No. 201, Sec. 2, Shipai Rd, Taipei, Taiwan ROC; 2 Department of Pathology, National Yang-Ming University School of Medicine, No. 155, Sec. 2, Linong St, Taipei, Taiwan ROC; 3 Institute of Biochemistry and Molecular Biology, National Yang-Ming University, No. 155, Sec. 2, Linong St, Taipei, Taiwan ROC; 4 Department of Life Sciences and Institute of Genome Sciences, National Yang-Ming University, 11221 No. 155, Sec. 2, Linong Street, Taipei, Taiwan ROC

**Keywords:** Rho-kinase, Metastasis, HER2, Estrogen Receptor, Progesterone receptor

## Abstract

**Background:**

Two isoforms of Rho-associated coiled-coil kinase (ROCK), ROCKI and ROCKII, play an important role in many cellular processes. Despite the accumulating evidence showing that ROCK could be a potential cancer therapeutic target, the relevant tumor types to ROCK activation are not well clarified. The aim of this study was to evaluate the ROCK activation status in different tumor types of breast cancer.

**Results:**

We evaluated the immunoreactivities of phosphorylation-specific antibodies of ROCKI and ROCKII to inform their kinase activation in 275 of breast carcinoma tissues, including 56 of carcinoma in situ, 116 of invasive carcinoma, and 103 of invasive carcinoma with metastasis. ROCKII activation signal detected in nucleus was significantly correlated with tumor metastasis, while ROCKI and cytosolic ROCKII activation signals made no significant difference in that metastasis. Furthermore, nuclear ROCKII activation signal was associated with poor clinical outcome and correlated with late tumor stage, low expression of estrogen receptor (ER) and progesterone receptor (PR), overexpression of human epidermal growth factor receptor 2 (HER2) and high Ki67 labeling index.

**Conclusions:**

Nuclear ROCKII activation signal might contribute to the tumor metastasis in breast cancer. Differences in ROCK activation that underlie the phenotypes of breast cancer could enhance our understanding for the use of ROCK inhibitors in cancer therapy.

## Background

The development of metastasis-specific therapy stratagem is an important issue for breast cancer since tumor metastasis is the main cause of breast cancer-related mortality [1, 2]. The small GTPases, RhoA and RhoC, are the key molecules in the invasive and metastatic cancer cell behaviors, as well as in tumor growth and cancer-associated alteration of extracellular matrix [3–6]. Rho-associated kinase (ROCK), a main effector of RhoA and RhoC, is a serine/threonine kinase and contributes to the stabilization of actin filaments and myosin-mediated contractility [7, 8]. Two ROCK isoforms, ROCKI (also known as ROKβ) and ROCKII (also known as ROKα), were identified [9, 10]. The two kinases have 65 % overall identity in humans with 87 % identity in the catalytic kinase domain [11]. It has been reported that activation of ROCK signaling increased tumor cell dissemination [12]. Inhibition of ROCK significantly reduced cell invasion and metastasis in several tumor models, such as breast carcinoma, hepatoma, melanoma, prostatic and lung cancers [13–17]. Application of ROCK inhibitor reduced cells metastasis in “human breast cancer metastasis to human bone” mouse model [18]. These data suggest that ROCK is involved in tumorigenesis and is a potential cancer therapeutic target. The combination of ROCK inhibitors with proteasome inhibitors in non-small-cell lung cancer and with tyrosine kinase inhibitors in chronic myeloid leukemia produced greater anti-cancer effects [19, 20]. Despite the significant effects of ROCK inhibition in many cancer studies, the clinical trials of ROCK inhibitors in cancer therapy is still limited since the relevance of tumor types to ROCK activation is not well clarified [11].

Breast cancer is a heterogeneous disease, including subtypes based on estrogen receptor (ER) and progesterone receptor (PR) status and amplification of human epidermal growth factor receptor 2 (HER2) [21]. The hormone receptor-positive cancers are the luminal A and B types. HER2-enriched type is identified as high expression of HER2 and low expression of ER/PR. Breast cancers with negative ER, PR, and HER2 status is “triple negative” and approximate the basal-like type [22, 23]. It is necessary to link the ROCK activation signals with specific subtypes. Although the high expression of ROCKs in cancer has been reported [17], it should be noted that the enhanced transcript or protein expression may not be necessarily correlated with the increase in their kinase activity. In our previous studies, we identified the autophosphorylation of ROCKI and ROCKII at the highly conserved Ser1333 and Ser1366 residues, respectively [24, 25]. We generated the phosphorylation-specific antibodies and validated their specificity by Western blot analysis combined with peptide competition and gene knockdown experiments. We also provided evidence that S1333 ROCKI and S1366 ROCKII phosphorylation can indicate their kinase active status in response to RhoA signaling [24, 25]. Thus, the kinase activation status of ROCKI and ROCKII in tissues could be evaluated directly by using these antibodies. The aim of this study was therefore to evaluate the ROCKI and ROCKII activation status in different tumor types of breast cancer, including carcinoma in situ (CIS), invasive carcinoma (IC) and invasive carcinoma with metastasis (ICM), by immunohistochemical staining with anti-pS1333 ROCKI and anti-pS1366 ROCKII antibodies. The differences of ROCK activation status that underlie the phenotypes of breast cancer were assayed, and their associations with clinicopathologic factors and clinical outcome were also characterized.

## Methods

### Study samples

Patients with primary breast carcinoma were retrieved from the surgical pathology file of the hospital from 1990 to 1999. The clinicopathological data including age, histologic type, grade, nodal status, stage at diagnosis, date of surgery, follow up data, and ER/PR/HER2 data were collected from the pathology and medical records. Overall survival was defined as the time from operation to death related to breast cancer. The study protocol was approved by the Institutional Review Board of Taipei Veterans General Hospital, Taiwan. In this retrospective study, the sample collection followed the ethical standards of the World Association’s Declaration of Helsinki and the need for informed consent was waived by the Institutional Review Board of the Taipei Veterans General Hospital, Taiwan.

### Tissue microarray (TMA) construction

All specimens were fixed in 10 % neutral buffered formalin. After reviewing the original histopathology slides for confirmation of the presence of the required tissues, two hundred and seventy-five tumor samples from patients with breast cancer were used in this study. The tumor samples according to their original diagnoses were classified as carcinomas in situ (CIS, *n* = 56), invasive carcinomas (IC, *n* = 116) and invasive carcinomas with metastasis (ICM, *n* = 103). Tumor samples larger than 1 cm, which were available and adequate for building tissue arrays with 2 mm tissue cylinders from 2 to 3 appropriate areas, were selected from each case. Two cores from representative areas of the tumors, or three cores from the tumors with heterogeneous features or those with available metastatic tumors were selected to construct tissue microarrays (TMAs). All patient identifiers were delinked from the tissues in the TMAs.

### Immunohistochemical staining and quantification

Tissue sections were immunostained using anti-pS1333 ROCKI and anti-pS1366 ROCKII antibodies on a Bond-max immunostainer (Leica Microsystems, Newcastle, UK). The production and validation of anti-pS1333-ROCKI and anti-pS1366-ROCKII antibodies have been described previously [24, 25]. Tissue sections were deparaffinized in xylene, rehydrated through serial dilutions of alcohol, and washed in phosphate-buffered saline (pH 7.2). On-board heat-induced antigen retrieval in pH 9.0 ethylenediamine tetraacetic acid (EDTA) for 30 min was performed. Sections were incubated with the primary antibodies (1: 750 for pS1333 ROCKI; 1:3200 for pS1366 ROCKII) for 60 min at room temperature. Visualization was performed using a VBS Refine polymer detection system (Leica Microsystems). ROCKII S1366 phosphopeptide or nonphosphopeptide (0.3 μg/ml) was added for the peptide competition experiment. All sections were counterstained with hematoxylin. Both nuclear staining and cytoplasmic staining of ROCKI and ROCKII phosphorylation were evaluated. The percentage of tumor cells with perceptible ROCK phosphorylation signal of in the nucleus was recorded for nuclear staining. Cytoplasmic staining was graded as negative/weak (no staining or <10 % faint staining), moderate (10–50 % area with intermediate staining), and strong (>50 % area with intense staining). Stains for ER (clone 6 F11, Leica Biosystems, Newcastle, UK, 1:100), PR (clone 16, Leica Biosystems, 1:150), HER2 (polyclone A0485, Dako, Glostrup, Denmark, 1:900) and Ki-67 (clone MIB-1, Dako, Glostrup, Denmark, 1:75) were performed. The evaluations of ER, PR, and HER2 followed previously reported instructions [26, 27]. One percent or more tumor cells exhibiting nuclear staining were regarded as positive for ER and PR [26]. HER2 positivity was defined by complete intense membrane staining in more than 10 % tumor cells [27]. The percentage of Ki67 positive tumor cells derived from four high-power fields (400×) was averaged for the Ki67 labeling index.

### Cell block preparation

HEK293T cells were maintained in Dulbecco's modified Eagle’s medium (DMEM) supplemented with 10 % (v/v) fetal bovine serum (FBS) in a humidified atmosphere of 5 % CO_2_/95 % air at 37 °C. For siRNA transfection experiments, 5 × 10^5^ of cells were transfected with or without of siRNA targeting human ROCKII (Dharmacon smartpool) by Lipofectamine 2000 reagent (Invitrogen, Carlsbad, CA). After 2 days, cells were than transfected with pEGFP-RhoAV14 by TurboFect reagent (Thermo Fisher Scientific) for 16 h. Cell were trypsinzied and collected by centrifugation at 900 rpm for 3 min. The cell pellets were fixed in 10 % neutral buffered formalin for 48 h, centrifuged and processed to paraffin cell block in the automatic tissue processor. A parallel set of cell lysate was prepared for the examination the protein expression levels of ROCKII and GFP-RhoAV14 by Western blot analysis with anti-ROCKII and anti-RhoA antibodies.

### Statistical analysis

Chi-square test for trend was used to compare the distributions of categorical variables. Differences between continuous variables were compared using the Mann–Whitney *U* test. Univariate Cox regression was performed for survival analyses. The survival curve was plot using Kaplan-Meier method. Their differences were compared by log-rank test. Multivariate Cox regression model was used to adjust the influence of significant prognostic factors. The statistical difference was considered significant when the *P* value was less than 0.05.

## Results

### Patient characteristics

The clinical and pathological characteristics including age, grade, ER/PR/HER2 status, and follow up of study cohort underlying the tumor type category are shown in Table 1.

**Table 1:** Clinical and pathological information of study cases

	Total	CIS	IC	ICM
Age (years)	53 (25, 95)	50 (25, 77)	54 (29, 95)	53 (28, 87)
Grade^a^	275	56	116	103
1	26 (9 %)	13 (23 %)	12 (10 %)	1 (1 %)
2	172 (63 %)	36 (64 %)	74 (64 %)	62 (60 %)
3	77 (28 %)	7 (13 %)	30 (26 %)	40 (39 %)
ER	266	54	111	101
Negative	88 (33 %)	7 (13 %)	37 (33 %)	44 (44 %)
Positive	178 (67 %)	47 (87 %)	74 (67 %)	57 (56 %)
PR	265	54	111	100
Negative	126 (48 %)	14 (26 %)	55 (50 %)	57 (57 %)
Positive	139 (52 %)	40 (74 %)	56 (50 %)	43 (43 %)
HER2	264	53	109	102
Negative	199 (72 %)	45 (85 %)	84 (77 %)	70 (69 %)
Positive	65 (25 %)	8 (15 %)	25 (23 %)	32 (31 %)
Follow up (months)	108 (1, 262)	97 (1, 213)	132 (9, 262)	86 (1, 257)

Data presented as mean (range) or number (%)

CIS, Carcinoma in situ; IC, Invasive carcinoma; ICM, Invasive carcinoma with metastasis; ER, estrogen receptor; PR, progesterone receptor. ^**a**^CIS was graded by nuclear grade; IC and ICM were graded by Nottingham histologic score

### Assessment of ROCK phosphorylation immunohistochemical (IHC) staining for breast carcinomas

The IHC results of ROCKI S1333 phosphorylation and ROCKII S1366 phosphorylation stratified by tumor classification are listed in Table 2. The status of ROCKI and ROCKII activation was determined by IHC staining with anti-pS1333 ROCKI and anti-pS1366 ROCKII antibody, respectively. Both ROCKI and ROCKII activation signals were observed in the cytoplasm and nucleus of tumor cells. The nuclear ROCKII signals were observed more frequently in the ICM 51/103 (50 %) cases than in the IC 35/116 (30 %) and CIS 11/56 (20 %) cases (*P* < 0.001). In addition, the percentage of tumor cells with perceptible ROCKII phosphorylation signal was evaluated, and the mean percentage of cells with nuclear signals was found to be significantly higher in ICM cases (20.8 %) than in IC (8.7 %) and CIS (6.9 %) cases (*P* = 0.003). ROCKI activation signal in nucleus was observed with no significant differences among CIS, IC or ICM cases (Table 2). Both ROCKI and ROCKII activation signals were observed in the cytoplasm with no significant differences among CIS, IC or ICM cases (Table 2), either. Overall, these data suggest that nuclear ROCKII activation signal is associated with tumor metastasis in invasive breast cancer.

**Table 2:** The results from ROCK phosphorylation staining, stratified by tumor types

Tumor type	Cytoplasmic staining				Nuclear staining	
	Negative/weak	Moderate	Strong	*P* ^a^	Mean % (Standard deviation)	*P* ^b^
ROCKII S1366 phosphorylation						
CIS	52 (93 %)	4 (7 %)	0 (0 %)	] 0.886 ] 0.814	6.9 (16.4)	] 0.329 ] 0.003
IC	107 (92 %)	9 (8 %)	0 (0 %)		8.7 (20.7)	
ICM	91 (88 %)	12 (12 %)	0 (0 %)		20.8 (29.6)	
ROCKI S1333 phosphorylation						
CIS	50 (89 %)	5 (9 %)	1 (2 %)	] 0.433 ] 0.450	1.3 (5.1)	] 0.908 ] 0.933
IC	96 (83 %)	19 (17 %)	0 (0 %)		0.9 (3.8)	
ICM	86 (83 %)	16 (16 %)	1 (1 %)		2.1 (8.4)	

Data presented as number (%) for cytoplasmic staining and mean % (Standard deviation) for nuclear staining. Bold values indicate statistical significance (*P* < 0.05)

CIS, Carcinoma in situ; IC, Invasive carcinoma; ICM, Invasive carcinoma with metastasis. ^**a**^Chi-square test for trend. ^**b**^Mann-Whitney test

### Validation the specificity of anti-pS1366 ROCK antibody in IHC staining

**Fig. 1:**
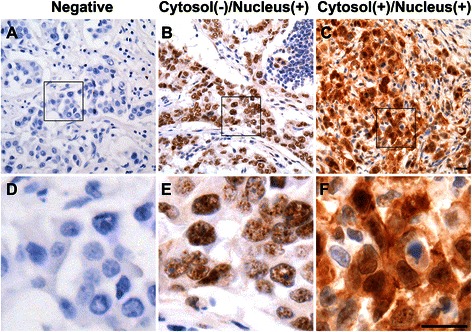
Examples of ROCKII S1366 phosphorylation immunohistochemical staining in breast carcinomas. Breast carcinoma cases varied greatly in ROCKII S1366 phosphorylation. **a**, **d**A grade-2 invasive ductal carcinoma with negative signal; (**b**, **e**) a metastatic ductal carcinoma in axillary lymph node showing significant positive signal in nuclei; (**c**, **f**) a grade-2 invasive ductal carcinoma with cytosol and nuclear positive signal. D-F shows enlargements of the boxed regions in A-C. Scale bars, 20 μm

**Fig. 2:**
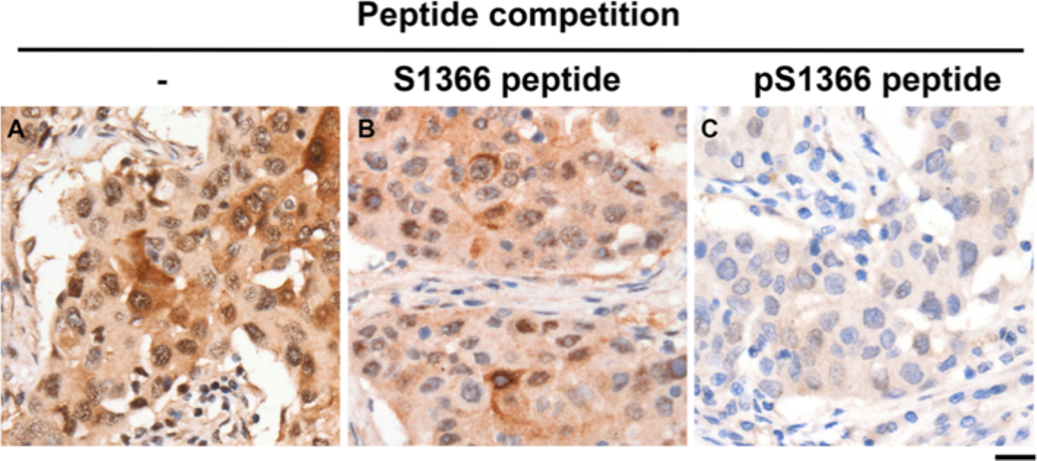
Competition of ROCKII S1366 phosphorylation immunohistochemical staining signal by peptide neutralization. Sample sections from one tumor of grade-3 invasive ductal carcinoma with axillary lymph node metastasis were stained with anti-pS1366 ROCKII antibody in the (**a**) absence or (**b**) presence of non-phosphorylated S1366 ROCKII peptide (0.3 μg/ml) or (**c**) phosphorylated S1366 ROCKII peptide (0.3 μg/ml). Scale bar, 20 μm

The representative IHC staining of ROCKII S1366 phosphorylation is showed in Figure 1. Three different breast carcinoma samples were shown and the ROCKII S1366 phosphorylation was detected negative or positive clearly at different proportional of nucleus and/or cytoplasm of tumor cells. To confirm the binding specificity of the anti-pS1366 ROCKII antibody, the ROCKII S1366 phosphorylated and non-phosphorylated peptides were used for antibody neutralization. The staining signal of a tumor sample revealing ROCII S1366 phosphorylation positive signal both in cytosol and nuclei could be abolished by competition with phosphorylated S1366 peptide but not by non-phosphorylated peptide, indicating the specificity of detection (Fig. 2).

**Fig. 3:**
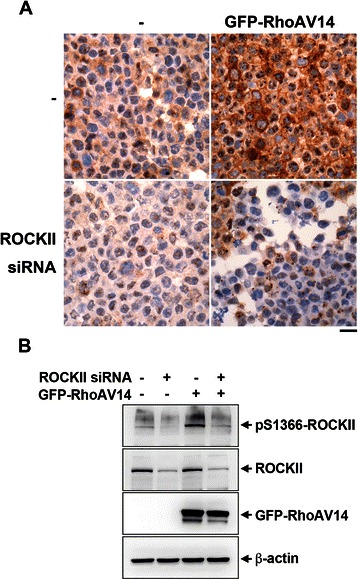
Validation of anti-pS1366 ROCKII antibody specificity in ROCKII depleted cell block. HEK293T cells were transiently transfected with or without siRNA targeting human ROCKII for 2 days and then transfected with or without pEGFP-RhoAV14 for 16 h. (**a**) Cells were pelleted and fixed. The samples from these cell blocks were stained with anti-pS1366 ROCKII antibody. Scale bar, 20 μm. (**b**) Cells were harvested for Western blotting with antibodies as indicated

To further validate the specificity, HEK293T cells were used to prepare cell blocks for IHC staining of ROCKII S1366 phosphorylation. We found that the ROCKII S1366 phosphorylation signal was significant increased by cells expression of constitutively active RhoAV14. Depletion of the endogenous ROCKII by siRNA transfection diminished the staining signal, confirming that the signal derived from ROCKII (Fig. 3a). Of note, the ROCKII S1366 phosphorylation level determined in cells without GFP-RhoAV14 transfection was so low that the effect of ROCKII depletion was inconspicuous. Enhancement of ROCKII activation by GFP-RhoAV14 expression highlights the decrease of ROCKII S1366 phosphorylation signal in knockdown cells. The ROCKII S1366 phosphorylation and protein expression of ROCKII and GFP-RhoAV14 were also confirmed by Western blot analysis (Fig. 3b).

### Association of nuclear ROCKII S1366 phosphorylation status with clinicopathologic features and clinical outcomes in invasive breast cancer

The positive staining of ROCKII activation signal in the invasive breast cancer (IC and ICM) respect to clinicopathologic features is listed in Table 3. The nuclear ROCKII S1366 phosphorylation signal was significantly stronger in tumors at advanced stage (*P* = 0.003), and correlated with ER negative (*P* = 0.002), PR negative (*P* = 0.017), HER2 positive status (*P* = 0.017) and high Ki67 labeling index (*P* = 0.044). There was no significant correlation between nuclear ROCKII activation signal and patient age (< 50 years vs. ≥ 50 years) or histological grade (Table 3). The IHC results of nuclear ROCKII S1366 phosphorylation stratified by molecular classification in invasive breast cancer are listed in Table 4. Our results show that nuclear ROCKII S1366 phosphorylation signal was significantly lower in the luminal types group compared to HER2-enriched group (*P* < 0.001) and to triple negative group (*P* = 0.044) (Table 4).

**Table 3:** ROCKII S1366 phosphorylation nuclear expressions in invasive breast cancers

	Number	Mean % (standard deviation)	*P* ^a^
Age (years)			
<50	90	13.6 (25.8)	0.706
≥ 50	129	14.9 (26.2)	
Grade			
1	13	2.7 (6.0)	] 0.220 ] 0.637
2	136	13.9 (25.5)	
3	70	17.5 (28.6)	
Stage			
I/II	147	9.8 (21.5)	0.003
III/IV	72	23.7 (31.5)	
ER			
Negative	131	23.3 (30.6)	0.002
Positive	81	9.0 (20.8)	
PR			
Negative	112	20.1 (30)	0.017
Positive	99	8.2 (18.6)	
HER2			
Negative	154	11.3 (23)	0.017
Positive	57	22.8 (31.4)	
Ki67			
< 20 %	34	5.9 (13.4)	0.044
≥ 20 %	36	17.8 (25.1)	

Bold values indicate statistical significance (*P* < 0.05). ER, estrogen receptor; PR, progesterone receptor. ^a^Mann–Whitney test

**Table 4:**
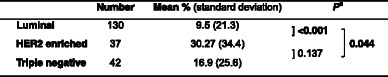
Stratify nuclear ROCKII S1366 phosphorylation by immunohistochemical subtypes in invasive breast cancers

Bold values indicate statistical significance (*P* < 0.05). ER, estrogen receptor; PR, progesterone receptor. ^a^Mann–Whitney test

**Fig. 4:**
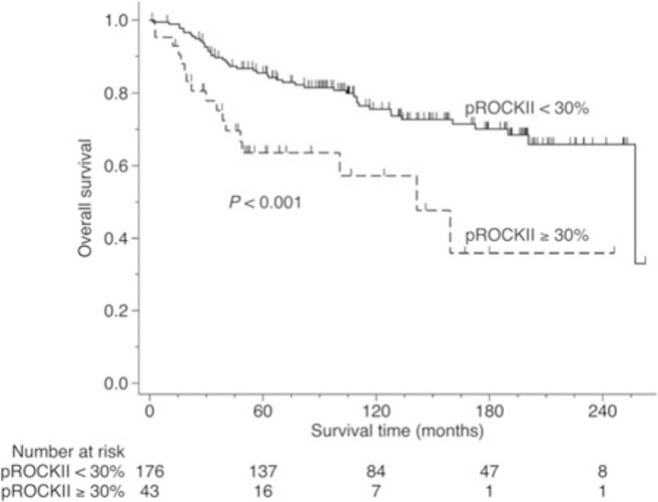
A high level of nuclear ROCKII activation was related to poor prognosis. Kaplan-Meier plot of the overall survival for 219 cases of invasive breast carcinoma dichotomized at the nuclear expression of pS1366 ROCKII of 30 %. The number of subjects at risk is listed above the X-axis. The difference is determined by log-rank test (*P* < 0.001)

Nuclear ROCKII activation signal was a significant prognostic factor, as revealed by univariate Cox regression analyses (Hazard ratio = 1.013, *P* = 0.004). The best cut-off value for the proportion of nuclear ROCKII activation to predict survival was 30 %; there was significant difference in the survival between cases with nuclear ROCKII activation signal ≥ 30 % (median survival 142 months) and cases with nuclear ROCKII activation signal < 30 % (median survival 257 months) (*P* < 0.001, Fig. 4). The relevance of nuclear ROCKII activation to other prognostic variables was then studied by multivariate analyses. Tumor stage was the most significant one among the prognostic variables studied such as patient age, histologic grade, tumor stage, ER, PR and HER2 status, and nuclear ROCKII activation (Table 5, model 1). Tumor stage is a complex function including tumor size, lymph node status and distant metastasis. Since nuclear ROCKII activation signal was significantly higher in ICM cases (Table 2) and associated with late tumor stage (Table 3), being highly dependent on tumor stage might confound the prognostic value of nuclear ROCKII activation. We then removed tumor stage form the multivariate analysis and found that the prognostic significance of nuclear ROCKII activation was revalidated (Hazard ratio = 2.116, *P* = 0.016; Table 5, model 2). This data further support the correlation of nuclear ROCKII activation with late tumor stage as well as metastasis.

**Table 5:** Multivariate Cox regression for cases with invasive carcinomas

	Hazard ratio (95 % CI)	*P*
Model 1		
Age (year)	1.019 (1.001, 1.037)	0.044
Grade		
1	1	
2	1.935 (0.258, 14.529)	0.521
3	1.062 (0.132, 8.565)	0.955
Stage		
I	1	
II	1.811 (0.673, 4.875)	0.240
III	6.144 (2.264, 16.677)	< 0.001
IV	58.450 (9.985, 342.165)	< 0.001
ER+	0.403 (0.169, 0.963)	0.041
PR+	1.768 (0.734, 4.262)	0.204
HER2+	1.302 (0.688, 2.463)	0.418
Nuclear pROCKII ≥ 30 %	1.414 (0.715, 2.794)	0.319
Model 2		
Age (year)	1.019 (1.000, 1.039)	0.050
Grade		
1	1	
2	3.244 (0.442, 23.801)	0.247
3	2.299 (0.297, 17.784)	0.425
ER+	0.563 (0.241, 1.316)	0.185
PR+	1.253 (0.532, 2.952)	0.605
HER2+	1.315 (0.713, 2.425)	0.381
Nuclear pROCKII ≥ 30 %	2.116 (1.152, 3.888)	0.016

Bold values indicate statistical significance (*P* < 0.05). ER, estrogen receptor; PR, progesterone receptor

## Discussion

ROCK plays a key role in multiple cellular activities primarily through its function on alteration of actin cytoskeleton dynamics [8, 28]. The importance of ROCK in pathogenesis is shown by using its specific inhibitors to interfere with disease progression in the clinical trials and animal experiments [29–32]. Recent studies have revealed a diverse range of functions of ROCK in cancer beyond its role in regulating cytoskeleton [11]. In this study, we observed the presence of ROCKII activation signal indicating by S1366 phosphorylation in a portion of cell nuclei, which seemed to associate with tumor metastasis and clinical outcome in the invasive breast cancer. However, it is still unknown whether nuclear ROCKII activation does contribute to tumor progression. We also observed the ROCKI and ROCKII activation signals in cytoplasm, although they showed no significant differences among different types of breast cancers. We cannot rule out the involvement of cytosolic ROCKI and ROCKII activation in tumor metastasis, because the spectrotemporal control of ROCK activation in cytoplasm might be very dynamic and not easy to evaluate in the fixed surgical samples.

In this study, we used the ROCKII S1366 phosphorylation signal to indicate its kinase activation regard our previous finding that S1366 was autophosphorylated once ROCKII is activated [24]. However, it cannot detect the ROCKII activation mediated by proteolytic cleavage of the inhibitory C-terminal region by granzyme B in apoptotic cells [33], as well as the somatic mutation which leading to premature termination of translation at Y1174 identified in a malignant melanoma cell line [34]. These truncated ROCKII are constitutive active and will not detected in our system. Moreover, we cannot route out the possibility that the increase of ROCKII S1366 might also contributed from other kinase in the cells. Therefore, S1366 phosphorylation may indicate the activation of full-length ROCKII but not absolutely equal to the overall status of ROCKII kinase activity in the cells.

Elevated transcripts or proteins levels of ROCKI and ROCKII have been reported breast cancer and other human cancers [17, 35–37]. However, it should be noted that increased gene expression might not be certainly correlate with their activation, since the ROCK is regulated by interaction with many specific regulatory molecules, both positively and negatively [38]. In this study, we found that ROCKII S1366 phosphorylation signal was detected in nucleus of the metastatic breast cancer. It implies that the ROCKII protein is localized at nucleus and a critical ROCKII activator is co-localized with nuclear compartmentalized ROCKII in metastatic breast tumors, such as nucleolar phosphoprotein NPM/B23 [39] and other Rho family members and their regulators can be present in nucleus [40–43]. In addition, it is also possible that a key ROCKII inhibitor is enhanced in cytoplasm of metastatic breast cancer cells. More studies are required to elucidate the molecular mechanisms of ROCKII activation in nucleus of metastatic breast cancer cells.

It is still an opened question about the function as well as down-stream substrate of ROCKII in the nucleus of metastatic breast cancer cells. Tanaka et al. have reported that ROCKII was localized in the nucleus and associated with transcriptional coactivator CBP/ p300 both in vitro and in vivo [44]. They confirmed the nuclear localization of ROCKII by immunofluorescence staining and nuclear extraction combined with gel filtration, and found that ROCKII was present in a large protein complex and partially co-localized with CBP/p300 in distinct insoluble nuclear structure. They also provided evidence that ROCKII phosphorylated CBP/p300 and increased its HAT activity in vitro, implying the contribution of nuclear ROCKII activation to gene regulation through CBP/p300. Several studies have revealed that CBP/p300 was related to tumorigenesis of various human cancers [45–47]. High expression of CBP/p300 in human breast cancer has been found to be correlated with tumor recurrence and predicts adverse prognosis [48]. The association of CBP/p300 with poor prognosis was also reported in other cancers [47, 49, 50]. In addition to the interaction with CBP/p300, it has been reported that ROCKII is translocated into nucleus to inhibit Cdc25A for cell cycle arrest in cells undergoing epithelial-mesenchymal transition stimulated by TGFβ [51]. The nuclear localization of Rho family members and their regulators are also reported [40–43]. Our finding of the correlation of nuclear ROCKII activation with tumor metastasis and poor prognosis in invasive breast cancer revealed a novel role of nuclear ROCKII activity in breast cancer. More experiments are needed to investigate the function of ROCKII in the nuclei of metastatic breast cancer cells.

## Conclusion

The importance of ROCK activation in cancer progression has been highlighted [11, 38]. In this study, we used anti-S1333 ROCKI and anti-S1366 ROCKII antibodies to inform the ROCKI and ROCKII kinase activation status in different types of breast cancer and found that ROCKII activation signal detected in nuclei was significantly correlated with tumor metastasis. We further found that nuclear ROCKII signal was negatively correlated with ER and PR expression and positively correlated with HER2 overexpression and high Ki67 labeling index in the invasive breast cancers. It was also associated with poor clinical outcome, which was relevant to advanced tumor stage. This is the first report on the relationship between ROCKII activation in nuclei and tumor metastasis as well as clinicopathologic features in invasive breast cancers.

## Abbreviations

CIS: carcinoma in situ
ER: estrogen receptor
HER2: human epidermal growth factor receptor 2
IC: invasive carcinoma
ICM: invasive carcinoma with metastasis
IHC: immunohistochemistry
PR: progesterone receptor
ROCK: Rho-associated kinase
TMA: tissue microarray

## References

[1] Rodenhiser DI, Andrews JD, Vandenberg TA, Chambers AF (2011). Gene signatures of breast cancer progression and metastasis. Breast Cancer Res.

[2] Steeg PS (2000). Molecular biology of breast cancer metastasis. 'Has it spread?': disarming one of the most terrifying questions. Breast Cancer Res.

[3] Ridley AJ (2001). Rho family proteins: coordinating cell responses. Trends Cell Biol.

[4] Ridley AJ (2001). Rho GTPases and cell migration. J Cell Sci.

[5] Etienne-Manneville S, Hall A (2002). Rho GTPases in cell biology. Nature.

[6] Yuan Z, Su J, You JF, Wang JL, Cui XL, Zheng J (2007). [Correlation of expression of RhoC with invasiveness of breast cancer cells *in vitro*]. Zhonghua Zhong Liu Za Zhi.

[7] Amano M, Fukata Y, Kaibuchi K (2000). Regulation and functions of Rho-associated kinase. Exp Cell Res.

[8] Riento K, Ridley AJ (2003). Rocks: multifunctional kinases in cell behaviour. Nat Rev Mol Cell Biol.

[9] Fujisawa K, Fujita A, Ishizaki T, Saito Y, Narumiya S (1996). Identification of the Rho-binding domain of p160ROCK, a Rho-associated coiled-coil containing protein kinase. J Biol Chem.

[10] Matsui T, Amano M, Yamamoto T, Chihara K, Nakafuku M, Ito M (1996). Rho-associated kinase, a novel serine/threonine kinase, as a putative target for small GTP binding protein Rho. EMBO J.

[11] Rath N, Olson MF (2012). Rho-associated kinases in tumorigenesis: re-considering ROCK inhibition for cancer therapy. EMBO Rep.

[12] Croft DR, Sahai E, Mavria G, Li S, Tsai J, Lee WM (2004). Conditional ROCK activation *in vivo* induces tumor cell dissemination and angiogenesis. Cancer Res.

[13] Ying H, Biroc SL, Li WW, Alicke B, Xuan JA, Pagila R (2006). The Rho kinase inhibitor fasudil inhibits tumor progression in human and rat tumor models. Mol Cancer Ther.

[14] Itoh K, Yoshioka K, Akedo H, Uehata M, Ishizaki T, Narumiya S (1999). An essential part for Rho-associated kinase in the transcellular invasion of tumor cells. Nat Med.

[15] Nakajima M, Hayashi K, Egi Y, Katayama K, Amano Y, Uehata M (2003). Effect of Wf-536, a novel ROCK inhibitor, against metastasis of B16 melanoma. Cancer Chemother Pharmacol.

[16] Somlyo AV, Bradshaw D, Ramos S, Murphy C, Myers CE, Somlyo AP (2000). Rho-kinase inhibitor retards migration and *in vivo* dissemination of human prostate cancer cells. Biochem Biophys Res Commun.

[17] Lane J, Martin TA, Watkins G, Mansel RE, Jiang WG (2008). The expression and prognostic value of ROCK I and ROCK II and their role in human breast cancer. Int J Oncol.

[18] Liu S, Goldstein RH, Scepansky EM, Rosenblatt M (2009). Inhibition of rho-associated kinase signaling prevents breast cancer metastasis to human bone. Cancer Res.

[19] Hamilton A, Gallipoli P, Nicholson E, Holyoake TL (2010). Targeted therapy in haematological malignancies. J Pathol.

[20] Kumar MS, Hancock DC, Molina-Arcas M, Steckel M, East P, Diefenbacher M (2012). The GATA2 transcriptional network is requisite for RAS oncogene-driven non-small cell lung cancer. Cell.

[21] Perou CM, Sorlie T, Eisen MB, van de Rijn M, Jeffrey SS, Rees CA (2000). Molecular portraits of human breast tumours. Nature.

[22] Schnitt SJ (2010). Classification and prognosis of invasive breast cancer: from morphology to molecular taxonomy. Modern pathology : an official journal of the United States and Canadian Academy oF Pathology, Inc.

[23] Goldhirsch A, Winer EP, Coates AS, Gelber RD, Piccart-Gebhart M, Thurlimann B (2013). Personalizing the treatment of women with early breast cancer: highlights of the St Gallen International Expert Consensus on the Primary Therapy of Early Breast Cancer 2013. Ann Oncol.

[24] Chuang HH, Yang CH, Tsay YG, Hsu CY, Tseng LM, Chang ZF (2012). ROCKII Ser1366 phosphorylation reflects the activation status. Biochem J.

[25] Chuang HH, Liang SW, Chang ZF, Lee HH (2013). Ser1333 phosphorylation indicates ROCKI activation. J Biomed Sci.

[26] Hammond ME, Hayes DF, Dowsett M, Allred DC, Hagerty KL, Badve S (2010). American Society of Clinical Oncology/College of American Pathologists guideline recommendations for immunohistochemical testing of estrogen and progesterone receptors in breast cancer. Arch Pathol Lab Med.

[27] Wolff AC, Hammond ME, Hicks DG, Dowsett M, McShane LM, Allison KH (2014). Recommendations for human epidermal growth factor receptor 2 testing in breast cancer: American Society of Clinical Oncology/College of American Pathologists clinical practice guideline update. Arch Pathol Lab Med.

[28] Van Aelst L, D'Souza-Schorey C (1997). Rho GTPases and signaling networks. Genes Dev.

[29] Mueller BK, Mack H, Teusch N (2005). Rho kinase, a promising drug target for neurological disorders. Nat Rev Drug Discov.

[30] Wettschureck N, Offermanns S (2002). Rho/Rho-kinase mediated signaling in physiology and pathophysiology. J Mol Med.

[31] Rikitake Y, Liao JK (2005). ROCKs as therapeutic targets in cardiovascular diseases. Expert Rev Cardiovasc Ther.

[32] Olson MF (2008). Applications for ROCK kinase inhibition. Curr Opin Cell Biol.

[33] Sebbagh M, Hamelin J, Bertoglio J, Solary E, Breard J (2005). Direct cleavage of ROCK II by granzyme B induces target cell membrane blebbing in a caspase-independent manner. J Exp Med.

[34] Greenman C, Stephens P, Smith R, Dalgliesh GL, Hunter C, Bignell G (2007). Patterns of somatic mutation in human cancer genomes. Nature.

[35] Liu X, Choy E, Hornicek FJ, Yang S, Yang C, Harmon D (2011). ROCK1 as a potential therapeutic target in osteosarcoma. J Orthop Res.

[36] Kamai T, Tsujii T, Arai K, Takagi K, Asami H, Ito Y (2003). Significant association of Rho/ROCK pathway with invasion and metastasis of bladder cancer. Clin Cancer Res.

[37] Vishnubhotla R, Sun S, Huq J, Bulic M, Ramesh A, Guzman G (2007). ROCK-II mediates colon cancer invasion via regulation of MMP-2 and MMP-13 at the site of invadopodia as revealed by multiphoton imaging. Lab Invest.

[38] Morgan-Fisher M, Wewer UM, Yoneda A (2013). Regulation of ROCK Activity in Cancer. J Histochem Cytochem.

[39] Ma Z, Kanai M, Kawamura K, Kaibuchi K, Ye K, Fukasawa K (2006). Interaction between ROCK II and nucleophosmin/B23 in the regulation of centrosome duplication. Mol Cell Biol.

[40] Balboa MA, Insel PA (1995). Nuclear phospholipase D in Madin-Darby canine kidney cells. Guanosine 5'-O-(thiotriphosphate)-stimulated activation is mediated by RhoA and is downstream of protein kinase C. J Biol Chem.

[41] Baldassare JJ, Jarpe MB, Alferes L, Raben DM (1997). Nuclear translocation of RhoA mediates the mitogen-induced activation of phospholipase D involved in nuclear envelope signal transduction. J Biol Chem.

[42] Tatsumoto T, Xie X, Blumenthal R, Okamoto I, Miki T (1999). Human ECT2 is an exchange factor for Rho GTPases, phosphorylated in G2/M phases, and involved in cytokinesis. J Cell Biol.

[43] Schmidt A, Hall A (2002). The Rho exchange factor Net1 is regulated by nuclear sequestration. J Biol Chem.

[44] Tanaka T, Nishimura D, Wu RC, Amano M, Iso T, Kedes L (2006). Nuclear Rho kinase, ROCK2, targets p300 acetyltransferase. J Biol Chem.

[45] Fan S, Ma YX, Wang C, Yuan RQ, Meng Q, Wang JA (2002). p300 Modulates the BRCA1 inhibition of estrogen receptor activity. Cancer Res.

[46] Bandyopadhyay D, Okan NA, Bales E, Nascimento L, Cole PA, Medrano EE (2002). Down-regulation of p300/CBP histone acetyltransferase activates a senescence checkpoint in human melanocytes. Cancer Res.

[47] Li M, Luo RZ, Chen JW, Cao Y, Lu JB, He JH (2011). High expression of transcriptional coactivator p300 correlates with aggressive features and poor prognosis of hepatocellular carcinoma. J Transl Med.

[48] Xiao XS, Cai MY, Chen JW, Guan XY, Kung HF, Zeng YX (2011). High Expression of p300 in Human Breast Cancer Correlates with Tumor Recurrence and Predicts Adverse Prognosis. Chin J Cancer Res.

[49] Gao Y, Geng J, Hong X, Qi J, Teng Y, Yang Y (2014). Expression of p300 and CBP is associated with poor prognosis in small cell lung cancer. Int J Clin Exp Pathol.

[50] Liao ZW, Zhou TC, Tan XJ, Song XL, Liu Y, Shi XY (2012). High expression of p300 is linked to aggressive features and poor prognosis of nasopharyngeal carcinoma. J Transl Med.

[51] Bhowmick NA, Ghiassi M, Aakre M, Brown K, Singh V, Moses HL (2003). TGF-beta-induced RhoA and p160ROCK activation is involved in the inhibition of Cdc25A with resultant cell-cycle arrest. Proc Natl Acad Sci U S A.

